# Supplementary figures and images for: TDP-43 directly inhibits mRNA accumulation in neurites through modulation of mRNA stability
Source: EMBO J. 2025 Dec 15;45(3):692–721. doi: 10.1038/s44318-025-00653-4 (PMC12864922; doi:10.1038/s44318-025-00653-4)

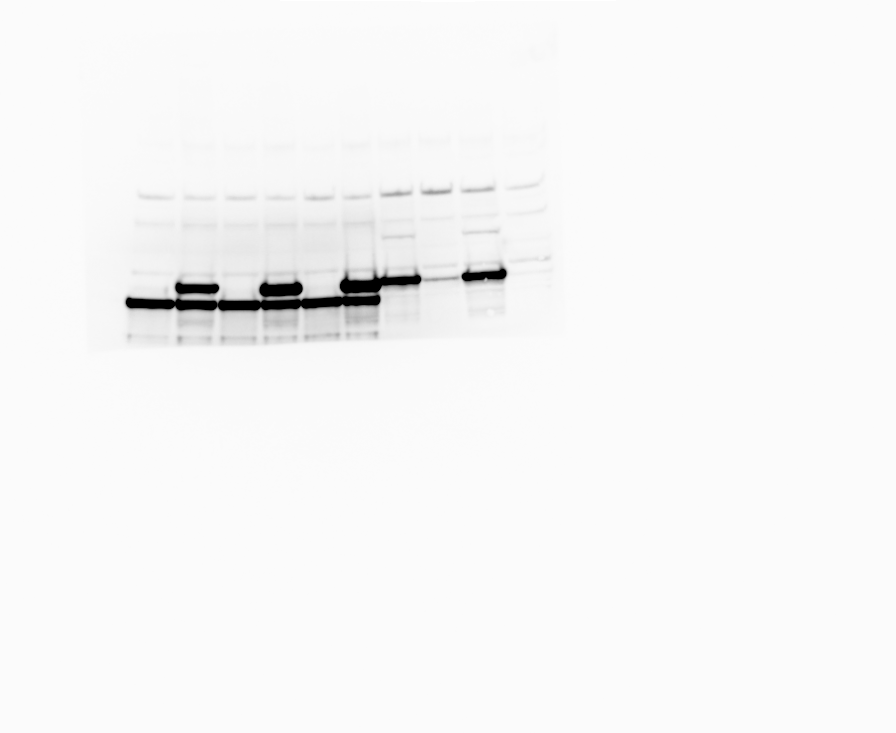

Supplement: Supplementary file 11 — Source data Fig. 1 [file 44318_2025_653_MOESM11_ESM.zip › Figure1/1B/2019-1024-ALSMut-TimeCourse-TDP43.tif]

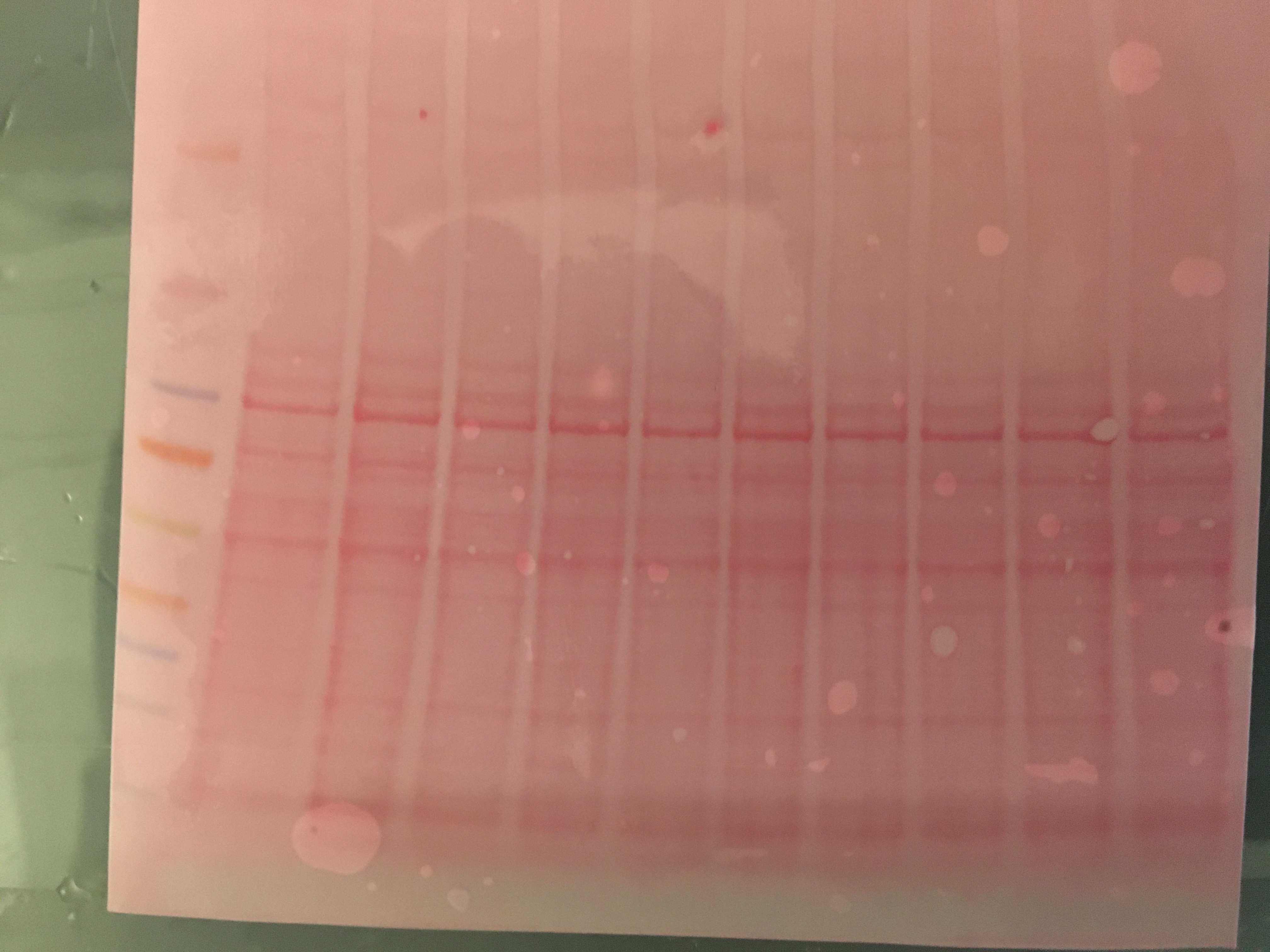

Supplement: Supplementary file 11 — Source data Fig. 1 [file 44318_2025_653_MOESM11_ESM.zip › Figure1/1B/10.24.19-Total Protein-Ponceau.JPEG]

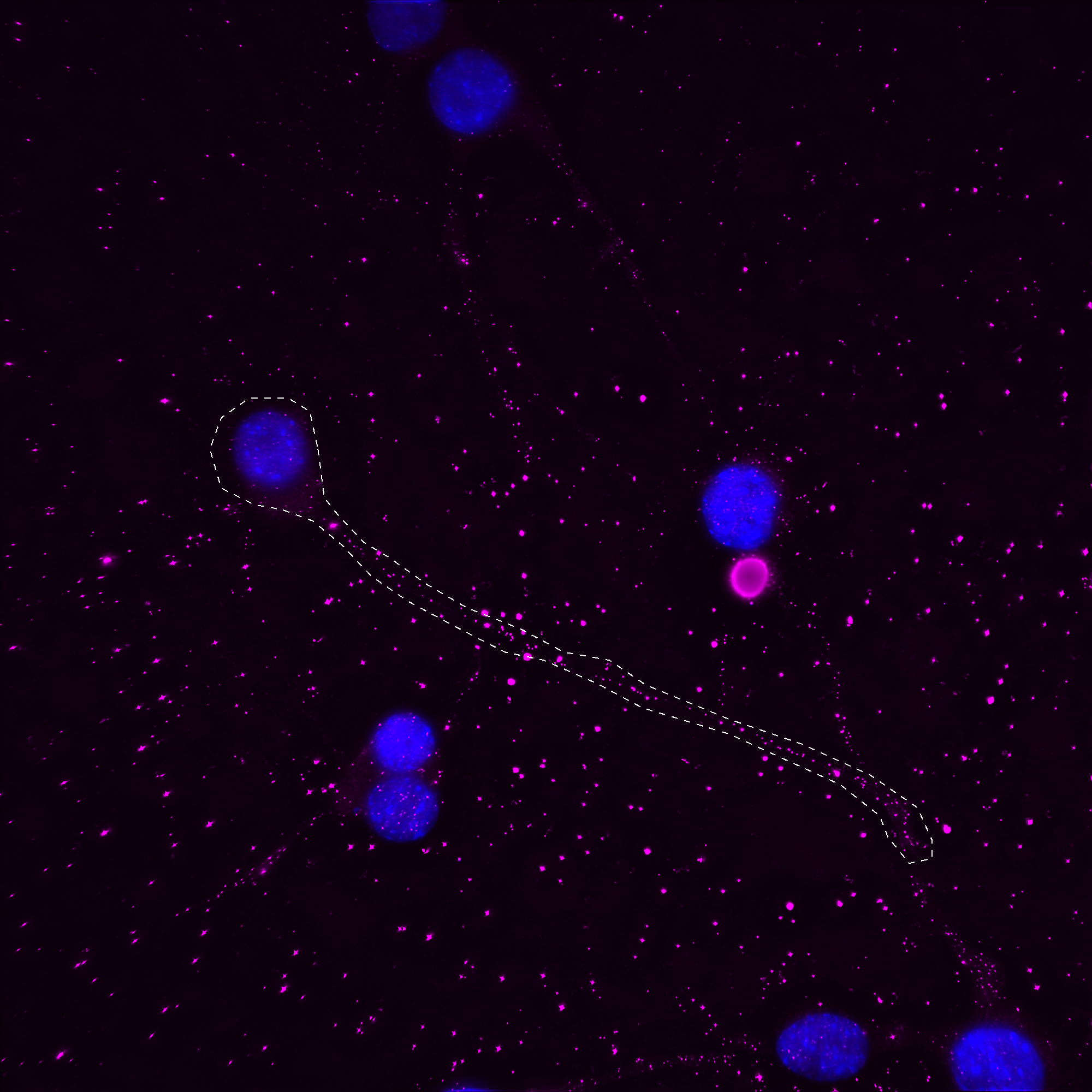

Supplement: Supplementary file 12 — Source data Fig. 2 [file 44318_2025_653_MOESM12_ESM.zip › Figure2/2A/KSR2_-DOX.tif]

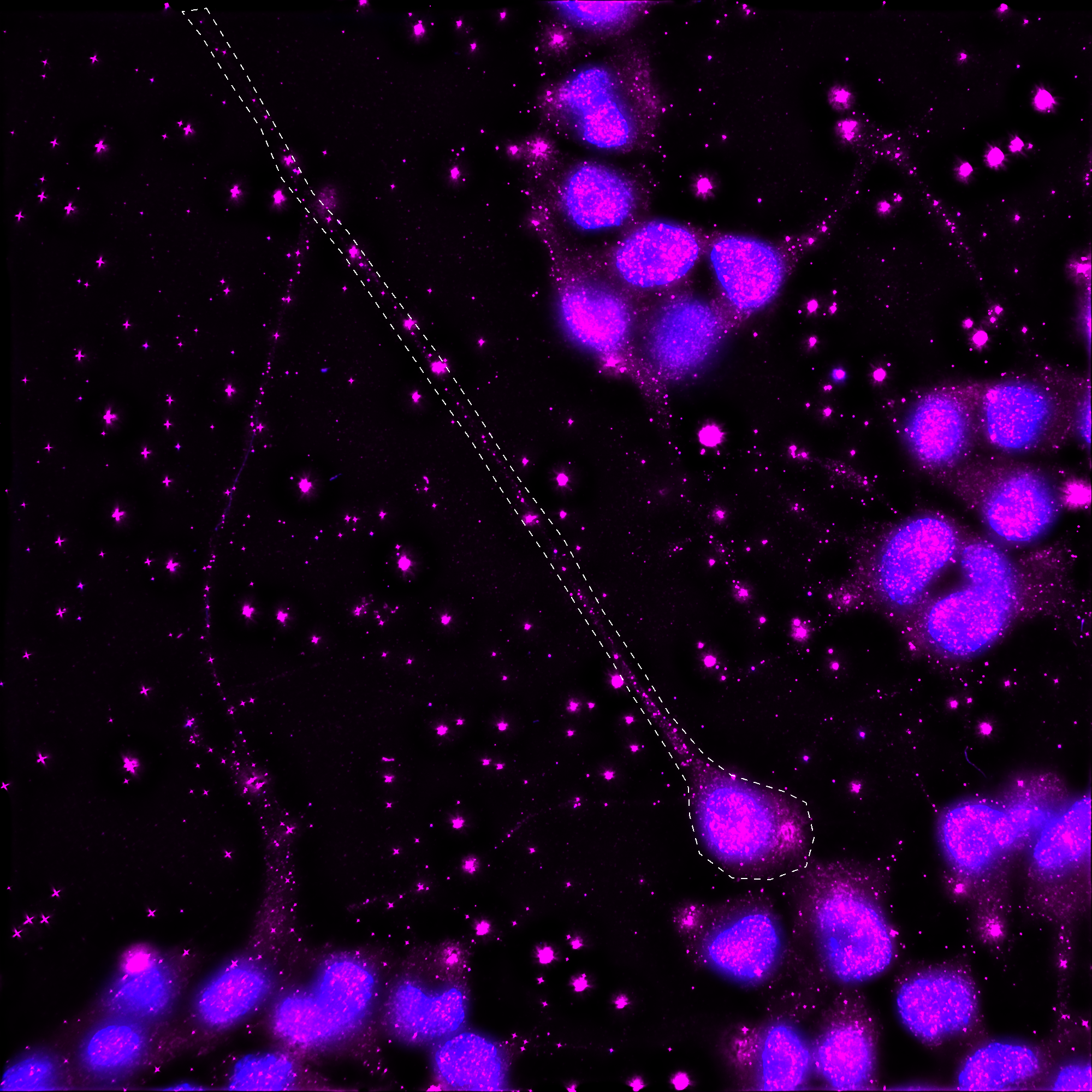

Supplement: Supplementary file 12 — Source data Fig. 2 [file 44318_2025_653_MOESM12_ESM.zip › Figure2/2A/KSR2_+DOX.tif]

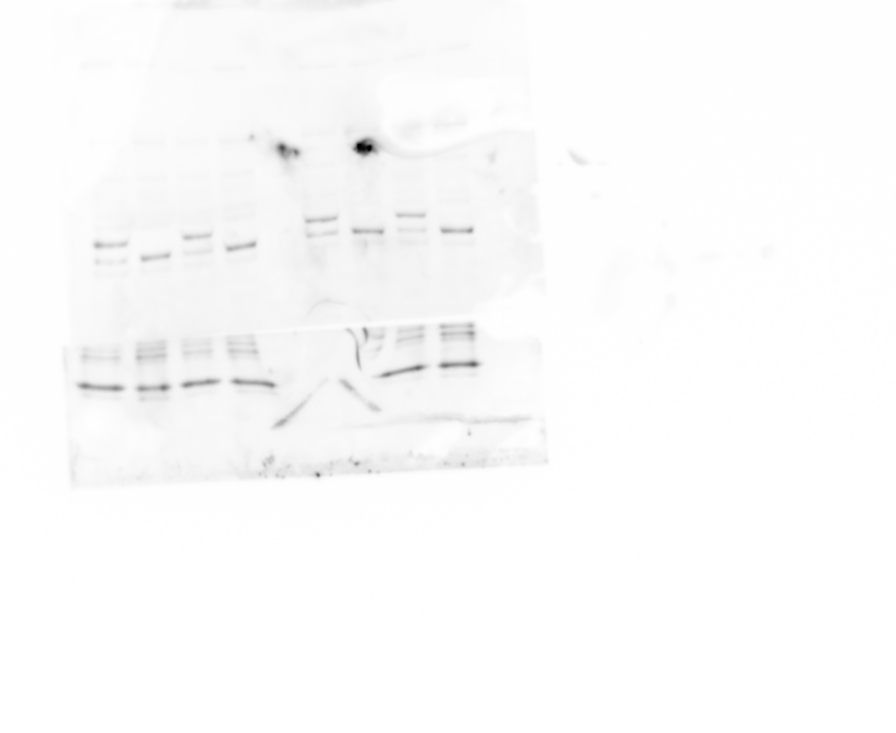

Supplement: Supplementary file 17 — Source data Fig. 7 [file 44318_2025_653_MOESM17_ESM.zip › Figure7/7C/24.01.23_16.36.10_S1_F04.tif]
