# Supplementary material for: TDP-43 directly inhibits mRNA accumulation in neurites through modulation of mRNA stability
Source: EMBO J. 2025 Dec 15;45(3):692–721. doi: 10.1038/s44318-025-00653-4 (PMC12864922; doi:10.1038/s44318-025-00653-4)
Supplement: Supplementary file 18 — Expanded View Figures [file 44318_2025_653_MOESM18_ESM.pdf]

Expanded View Figures

Figure EV1. Creation and characterization of TDP-43 knockout lines.

(**A**) Immunoblot of CAD cells with single copy, site-directed integration of doxycycline-repressible TDP-43. (**B**) Neurite lengths in CAD cells containing (−dox) or lacking (+dox) TDP-43. (**C**) Neurite enrichments of RNAs known to be neurite enriched, including those encoding ribosomal proteins and components of the electron transport chain. (**D**) PCA analysis of RNA expression values from soma and neurite samples of wild-type and TDP-43 knockout CAD cells. (**E**) As in (**D**), but for N2A cells. (**F**) Hierarchical clustering of RNA expression values from soma and neurite samples of wild-type and TDP-43 knockout CAD cells. (**G**) As in (**F**), but for N2A cells. (**H**) Changes in RNA localization to neurites in N2A cells upon loss of TDP-43. (**I**) Overlap in the identities of RNAs with increased neurite enrichments upon TDP-43 knockout in CAD and N2A cells. *P* values were calculated using a binomial test. (**J**) As in (**I**), but for RNAs with decreased neurite enrichments. *P* values were calculated using a binomial test. (**K**) Changes in neurite localization for *Ascl1*, *Diras1*, *Ksr2*, and *Wasf3* RNAs between wild-type and knockout CAD cells as measured by RT-qPCR. *P* values were calculated using a *t* test.

© The Author(s)

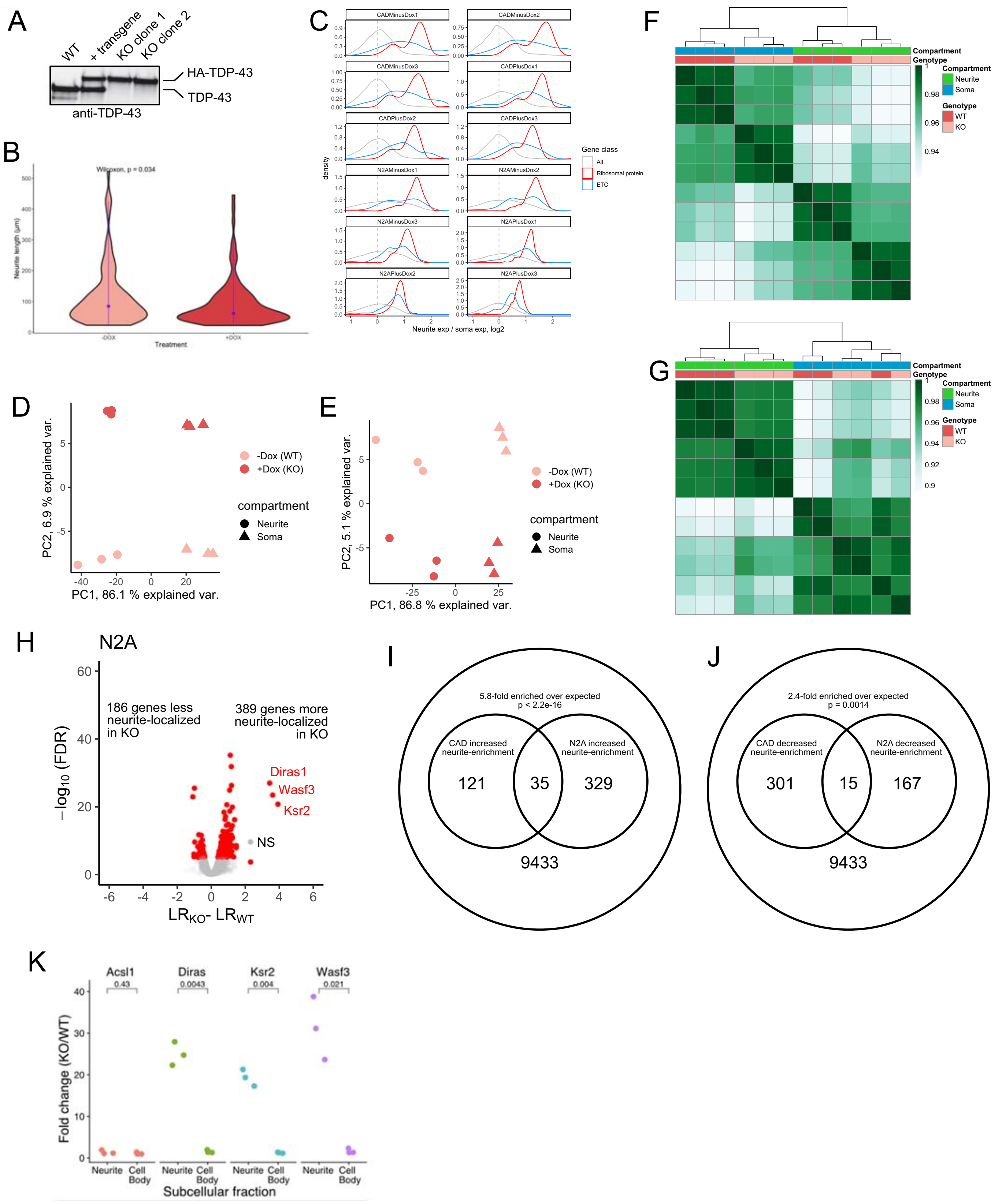
A
WT
+ transgene
KO clone 1
KO clone 2
HA-TDP-43
TDP-43
anti-TDP-43
B
Wilcoxon, p = 0.034
Neurite length (μm)
-DOX
+DOX
Treatment
C
CADMinusDox1
CADMinusDox2
CADMinusDox3
CADPlusDox1
CADPlusDox2
CADPlusDox3
N2AMinusDox1
N2AMinusDox2
N2AMinusDox3
N2APlusDox1
N2APlusDox2
N2APlusDox3
density
Neurite exp / soma exp, log2
Gene class
All
Ribosomal protein
ETC
D
PC2, 6.9 % explained var.
PC1, 86.1 % explained var.
-Dox (WT)
+Dox (KO)
compartment
Neurite
Soma
E
PC2, 5.1 % explained var.
PC1, 86.8 % explained var.
-Dox (WT)
+Dox (KO)
compartment
Neurite
Soma
F
Compartment
Genotype
Neurite
Soma
WT
KO
G
Compartment
Genotype
Neurite
Soma
WT
KO
H
N2A
186 genes less neurite-localized in KO
389 genes more neurite-localized in KO
Diras1
Wasf3
Ksr2
NS
−log10 (FDR)
LRKO- LRWT
I
5.8-fold enriched over expected
p < 2.2e-16
CAD increased neurite-enrichment
N2A increased neurite-enrichment
121
35
329
9433
J
2.4-fold enriched over expected
p = 0.0014
CAD decreased neurite-enrichment
N2A decreased neurite-enrichment
301
15
167
9433
K
Acsl1
0.43
Diras
0.0043
Ksr2
0.004
Wasf3
0.021
Fold change (KO/WT)
Neurite
Cell Body
Subcellular fraction

 © The Author(s)

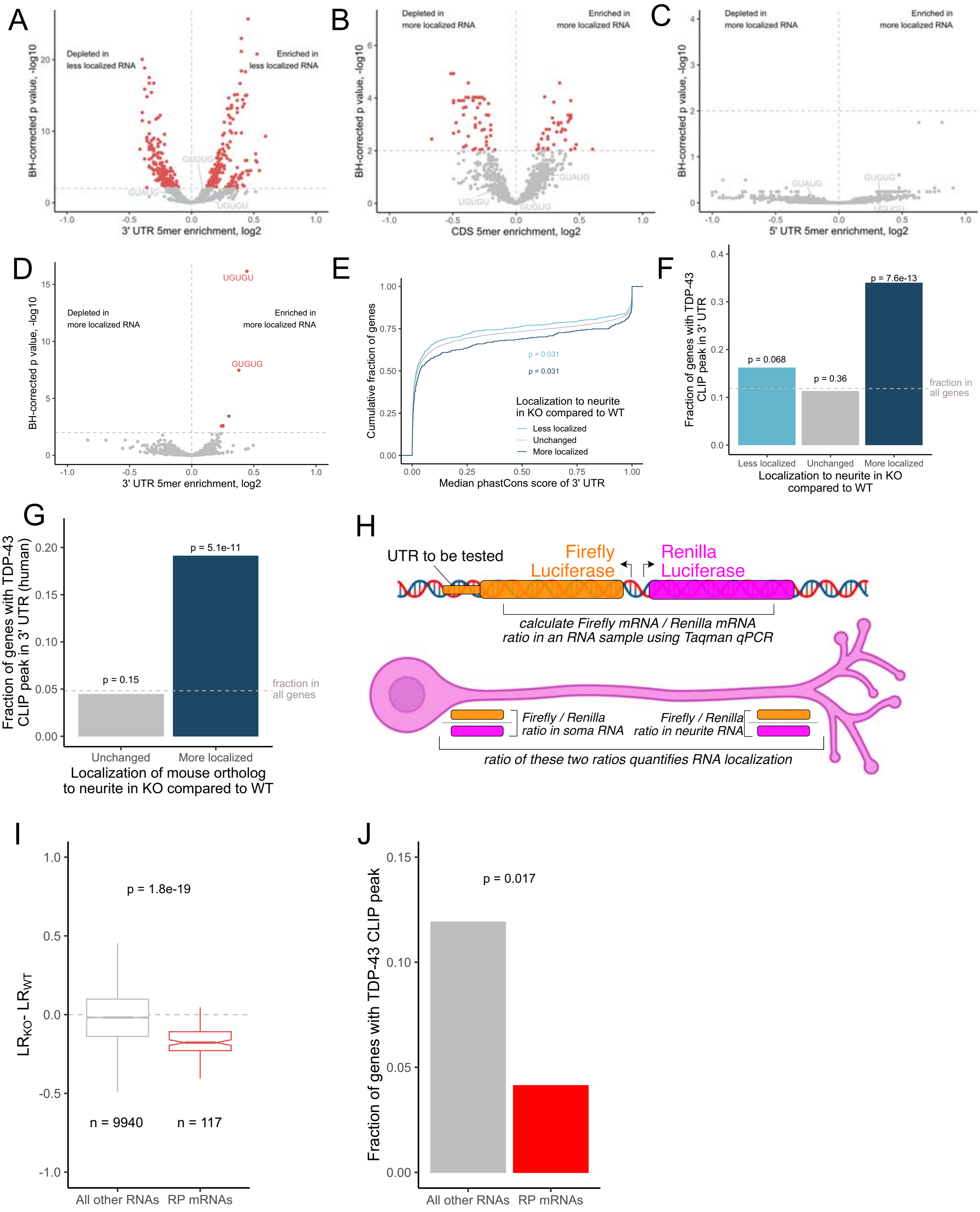
A
Depleted in less localized RNA
Enriched in less localized RNA
BH-corrected p value, -log10
3' UTR 5mer enrichment, log2
B
Depleted in more localized RNA
Enriched in more localized RNA
CDS 5mer enrichment, log2
C
5' UTR 5mer enrichment, log2
D
UGUGU
GUGUG
3' UTR 5mer enrichment, log2
E
Cumulative fraction of genes
p = 0.031
p = 0.031
Localization to neurite in KO compared to WT
Less localized
Unchanged
More localized
Median phastCons score of 3' UTR
F
Fraction of genes with TDP-43 CLIP peak in 3' UTR
p = 0.068
p = 0.36
p = 7.6e-13
fraction in all genes
Less localized
Unchanged
More localized
Localization to neurite in KO compared to WT
G
Fraction of genes with TDP-43 CLIP peak in 3' UTR (human)
p = 0.15
p = 5.1e-11
Unchanged
More localized
Localization of mouse ortholog to neurite in KO compared to WT
H
UTR to be tested
Firefly Luciferase
Renilla Luciferase
calculate Firefly mRNA / Renilla mRNA ratio in an RNA sample using Taqman qPCR
Firefly / Renilla ratio in soma RNA
Firefly / Renilla ratio in neurite RNA
ratio of these two ratios quantifies RNA localization
I
p = 1.8e-19
LR_{KO}- LR_{WT}
n = 9940
n = 117
All other RNAs
RP mRNAs
J
p = 0.017
Fraction of genes with TDP-43 CLIP peak
All other RNAs
RP mRNAs

© The Author(s)

Figure EV2. Analysis of sequences within RNAs whose localization is sensitive to TDP-43 loss.

(**A**) Enrichment for all 5mers in the 3′ UTRs of RNAs that were less neurite localized upon TDP-43 knockout in CAD cells ($n = 315$ UTRs) compared to the 3′ UTRs of RNAs whose localization was unaffected ($n = 9305$ UTRs). (**B**) Enrichment for all 5mers in the coding regions of RNAs that were more neurite localized upon TDP-43 knockout ($n = 160$ coding sequences) compared to the coding regions of RNAs whose localization was unaffected ($n = 9335$ coding sequences). (**C**) Enrichment for all 5mers in the 5′ UTRs of RNAs that were more neurite localized upon TDP-43 knockout ($n = 157$ UTRs) compared to the 5′ UTRs of RNAs whose localization was unaffected ($n = 9283$ UTRs). (**D**) Enrichment for all 5mers in the 3′ UTRs of the human orthologs of RNAs that were more neurite localized upon TDP-43 knockout ($n = 162$ UTRs) compared to the 3′ UTRs of the human orthologs of RNAs whose localization was unchanged ($n = 9237$ UTRs). (**E**) Phastcons scores for 3′ UTRs of RNAs with the indicated changes in neurite localization upon TDP-43 knockout. (**F**) Fraction of RNAs with CLIP-seq peaks in their 3′ UTRs for RNAs with the indicated changes in neurite localization upon TDP-43 knockout. (**G**) As in (**F**), but for the human orthologs of the genes in (**F**). (**H**) Schematic of reporter RNAs used in experiments monitoring RNA localization of reporters by RT-qPCR. (**I**) Differences in neurite localization between wild-type and knockout cells for all RNAs (left) or those encoding ribosomal proteins (right). (**J**) Fraction of genes with a TDP-43 in their 3′ UTR for all RNAs (left) or those encoding ribosomal proteins (right). *P* values for (**A–D**) were calculated using a Fisher's exact test. *P* values for (**E**, **I**) were calculated using a Wilcoxon rank-sum test. *P* values for (**F**, **G**, **J**), were calculated using a binomial test. For boxplots, centers represent medians, the bounds of boxes represent the 25th and 75th percentiles, and the tips of whiskers represent the value furthest from the bounds of the box that is no more than 1.5 times the interquartile range from the bounds of the box.

 © The Author(s)

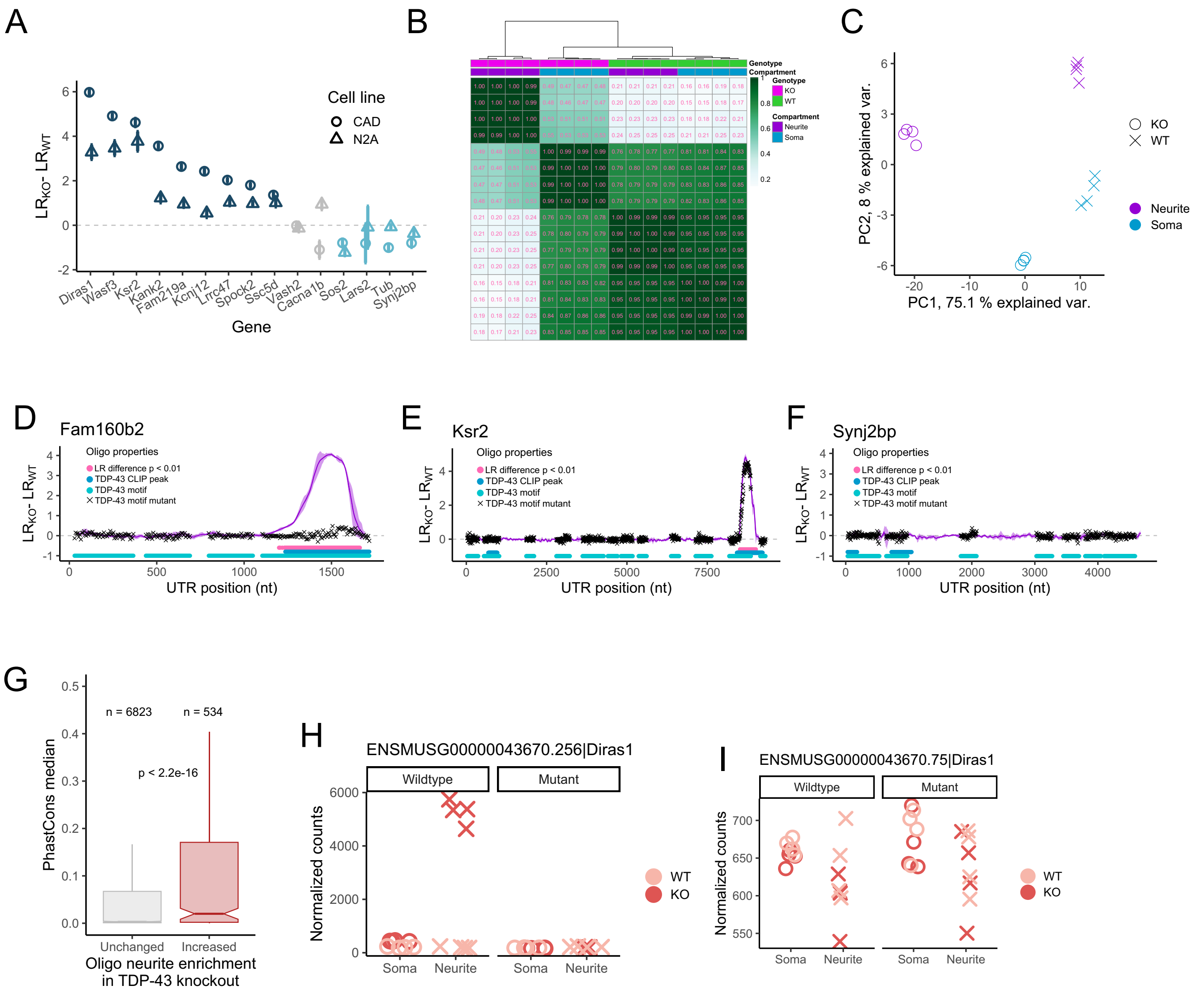

Figure EV3. Identification of sequences within 3′ UTRs that drive TDP-43-dependent RNA localization using a massively parallel reporter assay.

(**A**) Neurite enrichments for genes chosen for further analysis by MPRA. $n = 3$ for all comparisons. Error bars represent standard error around the mean. (**B**) Hierarchical clustering of normalized expression counts for all MPRA reporters. (**C**) PCA analysis of normalized expression counts for all MPRA reporters. (**D**) Differences in neurite enrichment between wild-type and knockout cells for all oligos that tile across the *Fam160b2* 3′ UTR. Oligos that contain a TDP-43 motif are represented by a light blue circle, those that contain a TDP-43 CLIP-seq peak are represented by a dark blue circle, and oligos with mutated TDP-43 motifs are represented by a black x. (**E**) As in (**D**), but for the *Ksr2* RNA. (**F**) As in (**D**), but for the non-TDP-43-target *Synj2bp* RNA. (**G**) Conservation levels for oligo sequences that were not sufficient to drive TDP-43-dependent changes in RNA localization (left) and those that were sufficient (right). (**H**) Example of one oligo that contained a TDP-43 motif and lied within a TDP-43 CLIP-seq peak. (**I**) Example of one oligo that contained a TDP-43 motif but did not lie within a TDP-43 CLIP-seq peak. *P* values were calculated using Wilcoxon rank-sum tests. For boxplots, centers represent medians, the bounds of boxes represent the 25th and 75th percentiles, and the tips of whiskers represent the value furthest from the bounds of the box that is no more than 1.5 times the interquartile range from the bounds of the box.

© The Author(s)

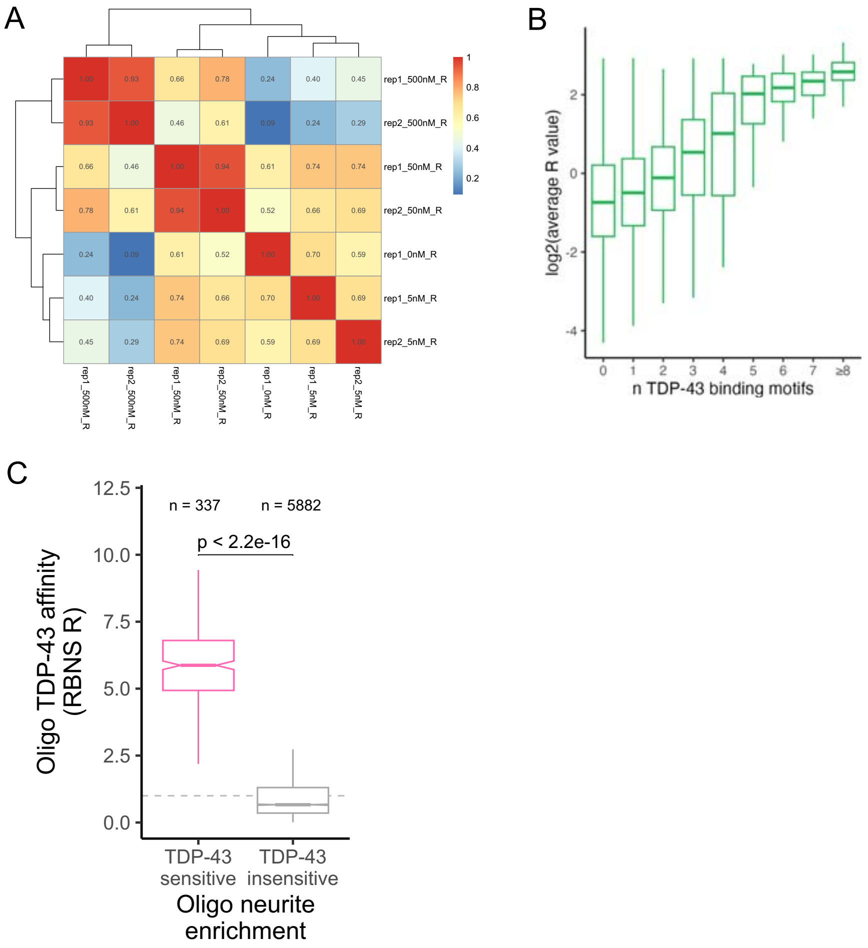

Figure EV4. Sequences that regulate RNA localization via TDP-43 are bound by purified recombinant TDP-43 in vitro.

(**A**) Hierarchical clustering of TDP-43 affinity values for all 260mer sequences as defined by RBNS. (**B**) Number of TDP-43 motifs in each 260mer sequence and the affinity of that sequence for TDP-43. Number of oligos in each motif bin: 0 = 6457, 1 = 2050, 2 = 652, 3 = 231, 4 = 69, 5 = 67, 6 = 73, 7 = 37, 8 + = 301. (**C**) Differences in TDP-43 affinity for RNA sequences that were sufficient to drive TDP-43-dependent changes in RNA localization (left) and those that were not (right). *P* values were calculated using Wilcoxon rank-sum tests. For boxplots, centers represent medians, the bounds of boxes represent the 25th and 75th percentiles, and the tips of whiskers represent the value furthest from the bounds of the box that is no more than 1.5 times the interquartile range from the bounds of the box.

 © The Author(s)

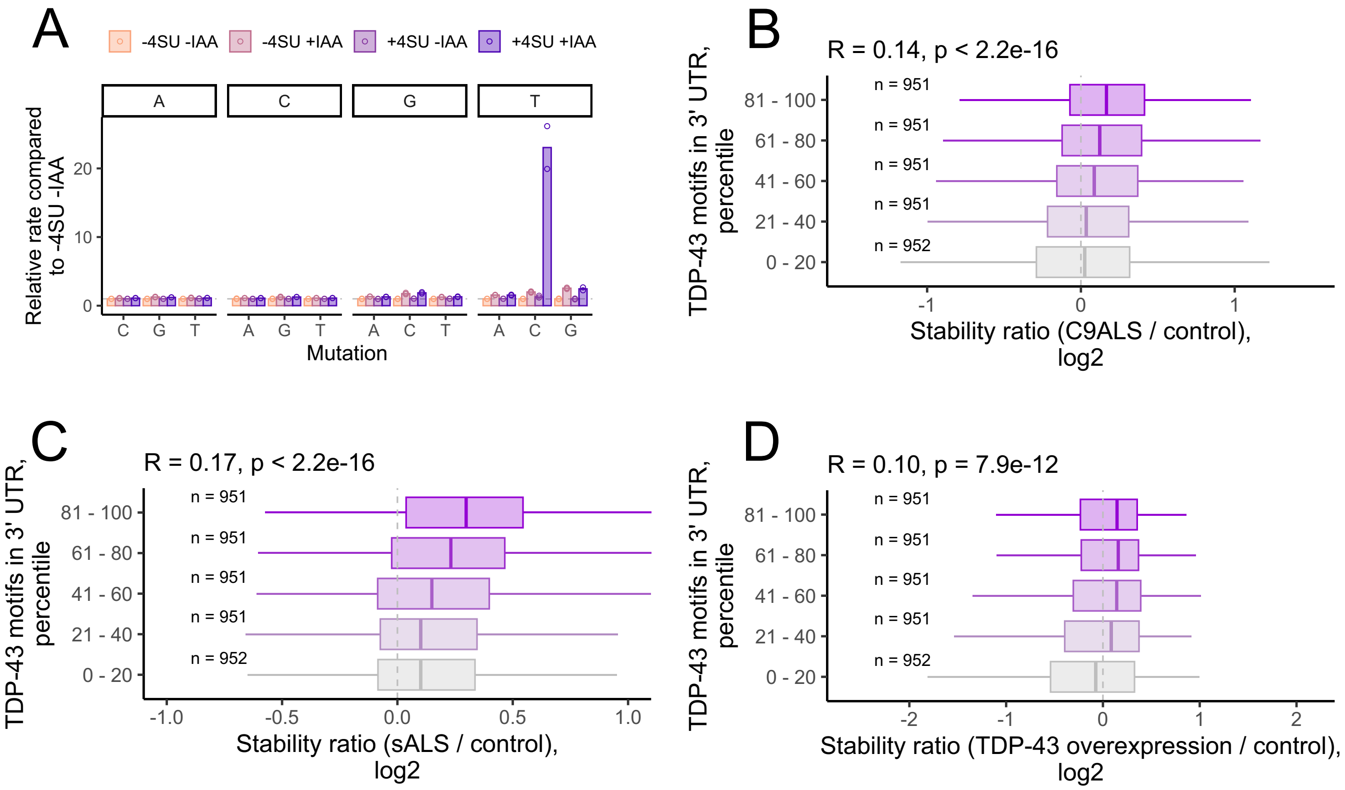

Figure EV5. TDP-43 motifs in 3′ UTRs are associated with an aberrant increase in RNA stability in disease settings.

(**A**) Nucleotide conversion rates for SLAM-seq samples containing or lacking 4SU and iodoacetamide (IAA). (**B**) Changes in stability as identified by BruChase-Seq between C9ALS and control samples for RNAs with the indicated TDP-43 motif content in their 3′ UTRs. (**C**) As in (**B**), but for changes in stability between sALS and control samples. (**D**) As in (**B**), but for changes in stability between TDP-43 overexpression and control samples. The indicated correlation coefficients and *P* values are from Spearman tests. For boxplots, centers represent medians, the bounds of boxes represent the 25th and 75th percentiles, and the tips of whiskers represent the value furthest from the bounds of the box that is no more than 1.5 times the interquartile range from the bounds of the box.

© The Author(s)

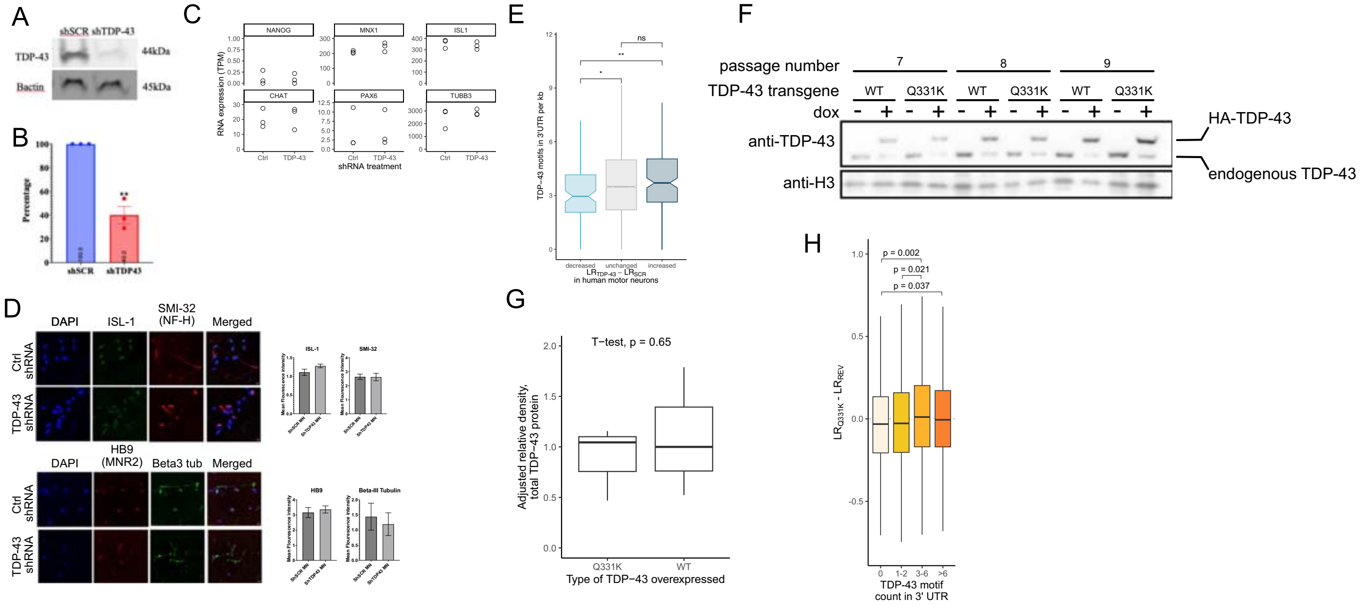

Figure EV6. In human motor neurons, TDP-43 knockdown is associated with increased neurite enrichment of RNAs containing TDP-43 motifs in their 3′ UTRs.

(**A**) Immunoblot of TDP-43 in human motor neuron samples treated with shRNA against TDP-43 or a control scrambled RNA. (**B**) Quantification of TDP-43 levels in TDP-43 knockdown experiments. Bars represent standard deviations. *P* values were calculated using a *t* test. ** represents $P < 0.01$. Exact *P* value 0.008. $n = 3$ biological replicates. Error bars represent standard deviation around the mean. (**C**) RNA expression levels of pluripotency and neuron marker genes in human motor neurons treated with control and TDP-43-targeting shRNAs. $n = 3$ biological replicates. (**D**) Immunofluorescence images and signal quantification for pluripotency and neuron marker genes in human motor neurons treated with control and TDP-43-targeting shRNAs. Bars represent standard deviations. (**E**) TDP-43 motif content in the 3′ UTRs of RNAs with the indicated changes in neurite localization in human motor neurons between samples treated with TDP-43 shRNA and those treated with control, scrambled shRNA. Exact p values: decreased vs. unchanged 0.015, decreased vs. increased 0.002, unchanged vs. increased 0.15. (**F**) Immunoblot of endogenous and transgenic TDP-43 levels with and without doxycycline-inducible transgene expression. (**G**) Relative amount of total TDP-43 protein, as quantified by immunoblotting, in CAD cells inducibly expressing integrated wild-type and Q331K TDP-43 transgenes. (**H**) Difference in neurite enrichment between TDP-43 Q331K and wild-type human motor neurons for RNAs with the indicated number of TDP-43 binding sites (defined as GUGUG, UGUGU, and GUAUG) in their 3′ UTRs. *P* values were calculated using Wilcoxon rank-sum tests. NS (not significant) represents $P > 0.05$, $^*P < 0.05$, $^{**}P < 0.01$. For boxplots, centers represent medians, the bounds of boxes represent the 25th and 75th percentiles, and the tips of whiskers represent the value furthest from the bounds of the box that is no more than 1.5 times the interquartile range from the bounds of the box.

 © The Author(s)
