# Supplementary material for: TDP-43 directly inhibits mRNA accumulation in neurites through modulation of mRNA stability
Source: EMBO J. 2025 Dec 15;45(3):692–721. doi: 10.1038/s44318-025-00653-4 (PMC12864922; doi:10.1038/s44318-025-00653-4)
Supplement: Supplementary file 1 — Peer Review File [file 44318_2025_653_MOESM1_ESM.pdf]

THE EMBO JOURNAL

TDP-43 directly inhibits mRNA accumulation in neurites through modulation of mRNA stability

Charlie Moffatt, Ankita Arora, Katherine Vaeth, Bryan Guzman, Gurprit Bhardwaj, Audrey Hoelscher, Levi Gifford, Holger Russ, Daniel Dominguez, and Matthew Taliaferro

Corresponding author(s): Matthew Taliaferro (matthew.taliaferro@cuanschutz.edu) , Daniel Dominguez (didoming@email.unc.edu)

Review Timeline:

Submission Date:	22nd Apr 25
Editorial Decision:	6th Jun 25
Revision Received:	13th Aug 25
Editorial Decision:	29th Sep 25
Revision Received:	9th Oct 25
Accepted:	29th Oct 25

Editor: Cornelius Schneider

Transaction Report:

(Note: With the exception of the correction of typographical or spelling errors that could be a source of ambiguity, letters and reports are not edited. Depending on transfer agreements, referee reports obtained elsewhere may or may not be included in this compilation. Referee reports are anonymous unless the Referee chooses to sign their reports.)

1st Editorial Decision 6th Jun 2025

Dear Dr. Taliaferro,

Thank you for submitting your manuscript for consideration by the EMBO Journal. It has now been seen by two referees whose comments are shown below.

Given the referees' positive recommendations, I would like to invite you to submit a revised version of the manuscript, addressing the comments of both reviewers. I should add that it is EMBO Journal policy to allow only a single round of revision, and acceptance of your manuscript will therefore depend on the completeness of your responses in this revised version.

When preparing your letter of response to the referees' comments, please bear in mind that this will form part of the Review Process File, and will therefore be available online to the community. For more details on our Transparent Editorial Process, please visit our website: https://www.embopress.org/page/journal/14602075/authorguide#transparentprocess

We generally allow three months as standard revision time. As a matter of policy, competing manuscripts published during this period will not negatively impact on our assessment of the conceptual advance presented by your study. However, we request that you contact the editor as soon as possible upon publication of any related work, to discuss how to proceed. Should you foresee a problem in meeting this three-month deadline, please let us know in advance and we may be able to grant an extension.

Thank you for the opportunity to consider your work for publication. I look forward to your revision.

Yours sincerely,

Cornelius Schneider, PhD
Editor
The EMBO Journal
c.schneider@embojournal.org

Instructions for preparing your revised manuscript:

Please make sure you upload a letter of response to the referees' comments together with the revised manuscript.

Please also check that the title and abstract of the manuscript are brief, yet explicit, even to non-specialists.

When assembling figures, please refer to our figure preparation guideline in order to ensure proper formatting and readability in print as well as on screen:
https://bit.ly/EMBOPressFigurePreparationGuideline
See also guidelines for figure legends: https://www.embopress.org/page/journal/14602075/authorguide#figureformat

At EMBO Press we ask authors to provide source data for the main manuscript figures. You will receive a separate email with instructions for providing source data with your revised manuscript, including how to upload and organize the files.

IMPORTANT: When you send the revision we will require
- a point-by-point response to the referees' comments, with a detailed description of the changes made (as a word file).
- a word file of the manuscript text.
- individual production quality figure files (one file per figure)
- a complete author checklist, which you can download from our author guidelines (https://www.embopress.org/page/journal/14602075/authorguide).
- Expanded View files (replacing Supplementary Information)
Please see out instructions to authors
https://www.embopress.org/page/journal/14602075/authorguide#expandedview
- a Reagents and Tools Table as part of the Methods section, which can be downloaded from our author guidelines (https://www.embopress.org/page/journal/14602075/authorguide#structuredmethods)

Please remember: Digital image enhancement is acceptable practice, as long as it accurately represents the original data and conforms to community standards. If a figure has been subjected to significant electronic manipulation, this must be noted in the figure legend or in the 'Materials and Methods' section. The editors reserve the right to request original versions of figures and the original images that were used to assemble the figure.

Further information is available in our Guide For Authors: https://www.embopress.org/page/journal/14602075/authorguide

We realize that it is difficult to revise to a specific deadline. In the interest of protecting the conceptual advance provided by the work, we recommend a revision within 3 months (4th Sep 2025). Please discuss the revision progress ahead of this time with the editor if you require more time to complete the revisions. Use the link below to submit your revision:

https://emboj.msubmit.net/cgi-bin/main.plex

Referee #1:

In this manuscript, Moffatt, Arora et al., investigate the TAR DNA binding protein 43 (TDP-43) and its role in RNA stability and the subsequent consequences in RNA localization to neurites in mouse CAD and N2A cell lines, human iPSC derived motor neurons as well as in-vitro reporter systems. Their data supports the conclusion that TDP-43 regulates localization through binding and destabilizing transcripts which are predominantly found in the soma. New findings include the mechanism by which TDP-43 retains transcripts in the cell soma and the discovery that ALS-associated mutations in TDP-43 result in a similar mislocalization of RNA, suggesting a potential pathological pathway in ALS patients. Overall, we found the study very interesting, with excellent data supporting the new findings. We think it is well-suited for the journal. We have some comments to improve the manuscript:

1. Fig. S1I, J: the overlap between the CAD and N2A cell lines is significant but very low. This needs to be discussed in the light of the rest of the paper.
2. Fig. S3B: "oligo abundance clustered by subcellular compartment then genotype", inconsistency: the figure shows clustering first by genotype and then by subcellular compartment.
3. Fig. S3E: for Ksr2, mutation of the motif did not influence localization, although it was in an iCLIP peak of TDP-43 binding. Since Ksr2 is one of the model genes, this should be discussed.
4. Fig. S1, legend: the reference to previous panels is incorrect: (E), as in D, not C; (G), as in F, not E and (J), as in I, not G.
5. Fig. 1C: more genes are less neurite-localized in KO than more neurite-localized. In contrast, by total abundance in Fig. 1E,F, RNAs seem more neurite-localized in KO. This should be further discussed or analyzed. For example, are less neurite-localized genes lower-expressed? Are the changes less drastic?
6. 'We also found that ribosomal proteins were significantly aberrantly depleted from neurite TDP-43 knockout cells', do the authors mean '[...] that mRNAs encoding ribosomal proteins..."?
7. Fig. 6A is not very intuitive. The legend for the meaning of pink and green lines ('old' and 'new' mRNAs) are missing as well as the information that a star (*) indicates a change from T>C.
8. Fig. S5. We acknowledge that RNA regulation in disease and overexpression samples can be complex. It should however be mentioned that the ALS samples in Fig. S3B-C are models for loss-of-function, and S5D is a model of overexpression. Does this overexpression actually lead to loss-of-function (through aggregation, i.e., less expression of TDP-43), or is this an assumption? It should be mentioned, and discussed, how TDP-43 loss-of-function and overexpression cause the same stabilization patterns in RNAs with TDP-43 motifs.
9. On Page 12: "than those than expected" is a syntax error.

Referee #2:

General summary and opinion

In this paper Moffatt et al. show that the RNA-binding protein TDP-43, linked to amyotrophic lateral sclerosis and other neurodegenerative diseases, negatively regulates localization of specific transcripts in neuronal projections. The authors also identify RNA sequences that mediate interaction with TDP-43 and are necessary and sufficient for localization. The work is mainly based on experiments in TDP-43 KO mouse neuronal cell lines, which represent the most convincing model, however the authors propose a conserved mechanism on the basis of sequence conservation and one experiment in human iPSC-derived motoneurons. Moreover, they propose a link with ALS from experiments in mouse neuronal cells expressing the TDP-43 Q331K pathogenic variant. Despite these results (figures S6 and 7) are less convincing, overall the work is significant and of interest for the field.

Major concerns
1. The results of the first part of the paper, which are quite solid, point to a loss-of-function mechanism. Since the same transcripts become neurite-enriched in both TDP-43 KO and Q331K cells, the authors conclude that this is a loss-of-function mutation. However, if this is the case, how could be explained the fact that exogenous TDP-43 Q331K downregulates the endogenous TDP-43 mRNA? TDP-43 Q331K should not be able to bind its 3′ UTR to induce degradation. Moreover, to show that the Q331K is a loss of function mutation, the authors should provide a positive control, e.g. incorporation of the cryptic

exons in STMN2 and UNC13A mRNAs. Quantification of the WB in Fig. 7C is necessary to show that the levels of TDP-43 are similar in WT and mutant lines.
2. Regarding the human model, knock-down of TDP-43 was performed during differentiation from day 11 on. Does it interfere with normal differentiation of the cells? If the cell population at day 19 is different in shSCR and shTDP-43, the differential localization might be a secondary effect. One possibility to overcome this issue could be IF (for a motoneuronal marker) combined to smFISH (for mislocalized mRNAs) to show that mislocalization occurs in motoneurons.
3. To support the link between target mRNAs mislocalization and ALS, the authors could use patient-derived iPSCs (with proper isogenic controls), differentiated to motoneurons, to validate their findings at least for a subset of targets (e.g. by smFISH).

Minor concerns
1. In some figure legends there is no mention of the statistical analysis (e.g. Fig. 1D, Fig. S1, Fig. S6).

Additional non-essential suggestions for improving the study

1. It would be interesting to analyze the mutational burden in the targets 3'UTRs from large-scale whole-genome sequencing studies in ALS (e.g. project MINE).

We wish to thank the reviewers for their interest in our work and for taking the time to provide constructive, actionable feedback that has made the manuscript better. We have provided point-by-point responses to each issue brought up by the reviewers below.

Referee #1:

In this manuscript, Moffatt, Arora et al., investigate the TAR DNA binding protein 43 (TDP-43) and its role in RNA stability and the subsequent consequences in RNA localization to neurites in mouse CAD and N2A cell lines, human iPSC derived motor neurons as well as in-vitro reporter systems. Their data supports the conclusion that TDP-43 regulates localization through binding and destabilizing transcripts which are predominantly found in the soma. New findings include the mechanism by which TDP-43 retains transcripts in the cell soma and the discovery that ALS-associated mutations in TDP-43 result in a similar mislocalization of RNA, suggesting a potential pathological pathway in ALS patients. Overall, we found the study very interesting, with excellent data supporting the new findings. We think it is well-suited for the journal. We have some comments to improve the manuscript:

1. Fig. S1I, J: the overlap between the CAD and N2A cell lines is significant but very low. This needs to be discussed in the light of the rest of the paper.

This is an interesting point. It's difficult to know why exactly the absolute number is low, but the fact that the overlap is strongly enriched over the null expectation and statistically significant ($p < 2.2e16$ and 5.8-fold enriched for RNAs that show increased neurite-enrichment upon KO and $p = 0.0014$ and 2.4-fold enrichment for RNAs that are less neurite-enriched) gives us strong confidence that the results are generally reproducible and generalizable. Further, the fact that the overlap is stronger for the RNAs with increased neurite enrichment is also consistent with this being the more dominant and direct effect of TDP-43 loss on RNA localization.

There may be peculiarities that are specific to each cell line. The two lines come from very different sources. CAD cells are brain-derived while N2A cells are derived from a neuroblastoma.

A section detailing this has been added to the text: When we compared results from the two cell lines, we found a modest but significant overlap in the identities of mislocalized RNAs (**Figure EV1I, J**). The relatively low absolute number of overlapping genes may be due to a variety of reasons including the different brain (CAD) and neuroblastoma (N2A) origins of the two cell lines, but the statistically significant overlap between the groups gave us confidence in the overall reproducibility and generality of the results.

2. Fig. S3B: "oligo abundance clustered by subcellular compartment then genotype", inconsistency: the figure shows clustering first by genotype and then by subcellular compartment.

The reviewer is correct. Thank you for catching this. This has been updated in the text: To ensure the quality of MPRA data, we performed PCA and hierarchical clustering analysis on the oligo abundance counts from each compartment and genotype. Oligo abundances clustered by genotype then subcellular compartment, indicating the data was of high quality (**Figures EV3B, C**).

3. Fig. S3E: for Ksr2, mutation of the motif did not influence localization, although it was in an iCLIP peak of TDP-43 binding. Since Ksr2 is one of the model genes, this should be discussed.

We, too, have been puzzled by this for quite some time. We did not comment on it because we could not offer a definite explanation. However, the reviewer is correct in that it likely does warrant comment anyway. This result has now been discussed further in the text: Interestingly, although the 3′ UTR of Ksr2 contained a peak

of activity coincident with a TDP-43 CLIP site, mutation of the TDP-43 motifs within this peak did not abrogate activity (Figure EV3E), suggesting that perhaps TDP-43 binds a cryptic motif at this location or is tethered to the site via another RBP.

4. Fig. S1, legend: the reference to previous panels is incorrect: (E), as in D, not C; (G), as in F, not E and (J), as in I, not G.

Thank you for catching these mistakes. They have been corrected.

5. Fig. 1C: more genes are less neurite-localized in KO than more neurite-localized. In contrast, by total abundance in Fig. 1E,F, RNAs seem more neurite-localized in KO. This should be further discussed or analyzed. For example, are less neurite-localized genes lower-expressed? Are the changes less drastic?

In general, the changes for the less neurite-localized RNAs are less drastic. This is visible from the volcano plot in figure 1C where there are several RNAs in the top right corner whose increase in neurite-localization upon KO is greater than 10-fold while none of the less-localized RNAs have effect sizes of this magnitude. This large effect size was one reason we first chose to focus on the more-localized RNAs.

We did look into if the more-/less-localized RNAs tend to be highly or lowly expressed. We found that RNAs whose localization changed upon TDP-43 knockout were more highly expressed than those whose localization was unchanged (**Reviewer figure 1**). This is likely due to increased expression levels increasing statistical power and thus making RNAs more likely to meet statistical thresholds (i.e. FDR < 0.01). However, we did not observe a difference in expression between the RNAs that became more-localized and those that became less localized (**Reviewer figure 1**).

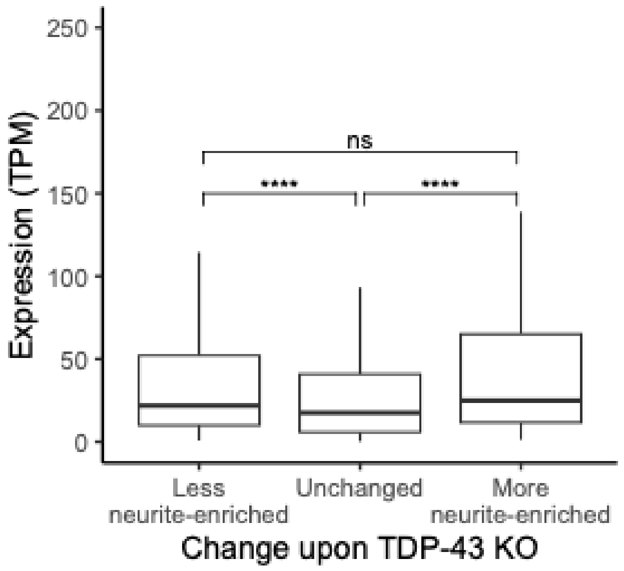

Reviewer figure 1. Expression levels of RNAs binned according to their change in neurite-enrichment upon loss of TDP-43. Statistical tests were performed using a Wilcoxon rank-sum test. **** denotes p < 0.001. ns denotes p > 0.05

6. 'We also found that ribosomal proteins were significantly aberrantly depleted from neurite TDP-43 knockout cells', do the authors mean '[...] that mRNAs encoding ribosomal proteins..."?

You are correct. Thank you for catching this. This has been corrected in the text.

7. Fig. 6A is not very intuitive. The legend for the meaning of pink and green lines ('old' and 'new' mRNAs) are missing as well as the information that a star (*) indicates a change from T>C.

Figure 6A has been redesigned with the requested information being added.

8. Fig. S5. We acknowledge that RNA regulation in disease and overexpression samples can be complex. It should however be mentioned that the ALS samples in Fig. S3B-C are models for loss-of-function, and S5D is a model of overexpression. Does this overexpression actually lead to loss-of-function (through aggregation, i.e., less expression of TDP-43), or is this an assumption? It should be mentioned, and discussed, how TDP-43 loss-of-function and overexpression cause the same stabilization patterns in RNAs with TDP-43 motifs.

This is an astute point. It is well established that even moderate overexpression of wildtype TDP-43 leads to cellular and organismal phenotypes that mimic those seen in ALS (e.g. neuropathies, neurodegeneration, motor deficits, and paralysis; PMID 40426231, PMID 20702714, PMID 35113871). This would be consistent with overexpression leading to an overall loss-of-function for TDP-43. How this might happen is not very well understood and is still a matter of debate among TDP-43 experts. It is worth noting, though, that overexpression of wildtype TDP-43 in mice is sufficient to bring about TDP-43-containing aggregates both in the nucleus and cytoplasm (PMID 20702714), which could plausibly lead to a loss-of-function similar to that seen in disease-associated samples.

We have updated the text to more clearly state the fact that wildtype TDP-43 overexpression can lead to aggregation and mimic a loss-of-function: TDP-43 can also be induced to aggregate by overexpressing it without its self-regulatory endogenous 3′ UTR (Ash *et al*, 2010; Wils *et al*, 2010). Even moderate overexpression of wildtype TDP-43 leads to TDP-43 aggregation as well as cellular and organismal phenotypes that mimic those seen in ALS (Yang *et al*, 2022; Xu *et al*, 2010).

9. On Page 12: "than those than expected" is a syntax error.

Thank you for catching this. This error has been fixed.

Referee #2:

General summary and opinion

In this paper Moffatt et al. show that the RNA-binding protein TDP-43, linked to amyotrophic lateral sclerosis and other neurodegenerative diseases, negatively regulates localization of specific transcripts in neuronal projections. The authors also identify RNA sequences that mediate interaction with TDP-43 and are necessary and sufficient for localization. The work is mainly based on experiments in TDP-43 KO mouse neuronal cell lines, which represent the most convincing model, however the authors propose a conserved mechanism on the basis of sequence conservation and one experiment in human iPSC-derived motoneurons. Moreover, they propose a link with ALS from experiments in mouse neuronal cells expressing the TDP-43 Q331K pathogenic variant. Despite these results (figures S6 and 7) are less convincing, overall the work is significant and of interest for the field.

Major concerns

1. The results of the first part of the paper, which are quite solid, point to a loss-of-function mechanism. Since the same transcripts become neurite-enriched in both TDP-43 KO and Q331K cells, the authors conclude that this is a loss-of-function mutation. However, if this is the case, how could be explained the fact that exogenous TDP-43 Q331K downregulates the endogenous TDP-43 mRNA? TDP-43 Q331K should not be able to bind its 3′ UTR to induce degradation. Moreover, to show that the Q331K is a loss of function mutation, the authors should provide a positive control, e.g. incorporation of the cryptic exons in STMN2 and UNC13A mRNAs. Quantification of the WB in Fig. 7C is necessary to show that the levels of TDP-43 are similar in WT and mutant lines.

This is an important point. There is published evidence that Q331K expression in mice does result in at least some splicing loss-of-function (PMID 23382207), that it results in the loss-of-function of TDP-43's role in promoting genome stability (PMID 31067307) and that it also results in the formation of TDP-43 aggregates (PMID 26108367). We have added these references to the text to further support this point: This suggests that Q331K TDP-43 is a partial loss-of-function mutation in the context of RNA localization, consistent with Q331K resulting in at least a partial loss-of-function of other TDP-43 activities (Arnold et al. 2013; Guerrero et al. 2019).

Our data in figure 7D would be consistent with Q331K acting generally as a *partial* loss-of-function allele with regards to RNA localization. This presumed partial loss-of-function would be expected to be less drastic than the complete knockout used in figures 1-6, and this is reflected in the relatively small effect size seen in figure 7D compared to what was observed in the knockout samples.

We agree that positive controls like the cryptic exons in STMN2 and UNC13A mRNAs would be a great place to further demonstrate that our Q331K expression system results in a loss of function. Unfortunately, though, those cryptic exons and the TDP-43 motifs that flank them are, to our knowledge, human-specific and are not conserved to mouse, meaning that we cannot do that analysis.

As an alternative approach, we compared global gene expression changes seen upon either TDP-43 knockout or inducible Q331K expression. We found that a relatively modest but highly significant positive correlation in overall gene expression changes between the two perturbations as might be expected when a total loss-of-function and a partial loss-of-function are compared ($R = 0.16$, $p < 2.2e-16$).

The reviewer brings up another great point in that if Q331K is a loss-of-function allele, how then is it able to regulate endogenous TDP-43 as we see in figure 7C? It should also be noted that the autoregulatory effect of TDP-43 is highly complex and acts at multiple levels including transcription, splicing, alternative polyadenylation (PMID 22855830), and RNA stability (PMID 21131904). It is possible that the effect of Q331K on some of these autoregulatory processes (e.g. transcription, splicing and alternative polyadenylation) may be less severe than its effect on RNA stability. If this was the case, Q331K would still be fully competent for autoregulation but would be deficient in its ability to regulate the stability (and therefore localization) of other targets. We fully admit that we have not tested this, though. We propose that after 7 days of Q331K expression, there is some loss of TDP-43's ability to regulate RNA stability that manifests as modest changes in RNA localization that mimic, although with much smaller effect sizes, what was seen with the TDP-43 knockout. However, because it is not a complete loss-of-function, Q331K is still competent to exert a downregulatory effect on endogenous TDP-43.

We also agree that quantification of the degree of autoregulation by the transgenic wildtype and Q331K alleles and the overall levels of TDP-43 between the samples in figure 7C is necessary. We have performed this and added the results as figures EV6F and EV6G.

2. Regarding the human model, knock-down of TDP-43 was performed during differentiation from day 11 on. Does it interfere with normal differentiation of the cells? If the cell population at day 19 is different in shSCR and shTDP-43, the differential localization might be a secondary effect. One possibility to overcome this issue could be IF (for a motoneuronal marker) combined to smFISH (for mislocalized mRNAs) to show that mislocalization occurs in motoneurons.

This is a good point. To get at this, we quantified the expression of several pluripotency (NANOG) and neuronal (Neurofilament-H, ISL-1, MNR2, TUBB3, CHAT, MNX1, PAX6) markers at the RNA level via RNAseq and at the protein level by immunofluorescence in the TDP-43 knockdown and control knockdown samples. In all cases, we did not observe any meaningful difference in marker expression between the control and TDP-43 knockdown samples. We therefore do not have evidence that differentiation is substantially differentially efficient between these samples. These results have been added as figures EV6C and EV6D.

3. To support the link between target mRNAs mislocalization and ALS, the authors could use patient-derived iPSCs (with proper isogenic controls), differentiated to motoneurons, to validate their findings at least for a subset of targets (e.g. by smFISH).

Great idea! To do this, instead of using patient-derived cells, we obtained engineered iPSC lines from the iPSC Neurodegenerative Disease Initiative (iNDI) via the Jackson Laboratory. We obtained two lines: one where the Q331K mutation had been engineered with Cas9 into both alleles at the TDP-43 locus as well as a control in which the mutation was re-engineered back to wildtype. We reasoned that this would provide a cleaner system than ALS patient-derived cells. We then differentiated these cells to motor neurons, and assayed RNA localization via subcellular fractionation and RNAseq as before.

Reminiscent of our results with the CAD TDP-43 knockout (figure 1), TDP-43 knockdown in human motor neurons (figure S6E), TDP-43 knockdown in mouse dorsal root ganglia (figure 6B), and the CAD Q331K transgene (figure 6D), we found a positive correlation between the number of TDP-43 motifs in an RNA's 3' UTR and the change in its localization to neurites following TDP-43 perturbation. Specifically, we found that RNAs that became more neurite-enriched in Q331K cells relative to controls had significantly more TDP-43 motifs in their 3' UTRs than expected. These results are now included as figure EV6H.

Although the results were statistically significant, the effect size was modest, perhaps due to the complexity of the iPS system relative to the CAD cells line and a relatively weak effect due to a potential partial loss of function of the Q331K allele relative to a full knockout.

We believe these results are consistent both with Q331K being a partial loss of function (at least as far as RNA localization is concerned), and with RNA mislocalization potentially occurring in ALS samples.

Minor concerns

1. In some figure legends there is no mention of the statistical analysis (e.g. Fig. 1D, Fig. S1, Fig. S6).

Thank you for catching these. Information regarding the statistical analysis for each of these figures has been added to the figure legends.

Additional non-essential suggestions for improving the study

1. It would be interesting to analyze the mutational burden in the targets 3'UTRs from large-scale whole-genome sequencing studies in ALS (e.g. project MINE).

This is a fantastic idea! It’s unclear to us whether the mutation of a single TDP-43 motif in the 3’ UTR of any one TDP-43 target RNA will be enough to be associated with ALS phenotypes, although correcting expression defects of specific splicing targets (e.g. STMN2 as the reviewer noted above) has rescued some phenotypes. This is something we are interested in pursuing, but view it as the start of a new project rather than something within the scope of the current one.

Dear Dr. Taliaferro,

Thank you for submitting a revised version of your manuscript. Your study has now been seen by all original referees, who find that their previous concerns have been addressed and now recommend publication of the manuscript. There remain only a few mainly editorial points that have to be addressed before I can extend formal acceptance of the manuscript:

- On the abstract page of the manuscript, please include 4-5 general keyword terms to enhance searchability.

- Please rename the Conflict of Interest section into "Disclosure and Competing Interests Statement", in accordance with our updated Guide to Authors (https://www.embopress.org/competing-interests)

- As we are switching from a free-text author contribution statement towards a more formal statement based on Contributor Role Taxonomy (CRediT) terms, please remove the present Author Contribution section and instead specify each author's contribution(s) directly in the Author Information page of our submission system during upload of the final manuscript. See https://casrai.org/credit/ for more information.

- Please check the FIGURE CALLOUTS: There are callouts for the figures that are not uploaded: Figure S2F; S3F; S3B, C; S3D-F, H; S3I; S6F,G => these should probably be callouts for EV figures

- Please update the DATASET EV LEGENDS: source file names, titles, legends and manuscript callouts all need to be updated to Dataset EV1-EV6 instead of Supplementary Table 1-6, legends should be removed from ms and uploaded as a separate tab/sheet in each Excel file

- Please remove the Reagent and Tools Table from the manuscript file and upload it as an individual file. For more information, please check https://www.embopress.org/page/journal/14602075/authorguide#structuredmethods and download the template for Reagent Table (attached for your convenience)

- Please provide suggestions for a short 'blurb' text prefacing and summing up the conceptual aspect of the study in two sentences (max. 250 characters), followed by 3-5 one-sentence 'bullet points' with brief factual statements of key results of the paper; they will form the basis of an editor-written 'Synopsis' accompanying the online version of the article. Please also provide an altered synopsis image, making sure that the aspect ratio conforms to our website's format - it should be exactly 550 pixels wide and between 300-600 pixels high.

- Please adjust the order of the manuscript sections: Title page with complete author information, Abstract, Keywords, Introduction, Results, Discussion, Methods, Data Availability Section, Acknowledgements, Disclosure and Competing Interests Statement, References, Main figure legends, Tables, Expanded Figure Legends.

- Please provide the specific URL for GSE288185 dataset in the data availability statement.

- Figure Legends (main + EV):
1. Please note that the exact p values are not provided in the legends of figures 3C, D; 4C, D, E, F, H; 5B, C; 6B-E; 7A, B, D; EV3 G, EV4 C, EV5 B, EV6 B, C

2. Please indicate the statistical test used for data analysis in the legends of figures 3B, EV2 A-D

3. Please note that the box plots need to be defined in terms of minima, maxima, centre, bounds of box and whiskers, and percentile in the legends of figures 3C, 4D E, F, H; 5B-D; 6B-E; 7A, B, D; EV2 I, EV3 G; EV4 B, C; EV5 B-D; EV6 C

4. Please note that information related to n is missing in the legends of figures 1D, 3B, C, D, E, F, G, H; 5B-D; 7A, B, D; EV1 H, EV2 A, B, C, D, I; EV3 A, EV3 G, EV4 B, C; EV5 B-D; EV6B, C

5. Please note that the error bars are not defined in the legend of figure EV3 A

6. Please note that the measure of center for the error bars needs to be defined in the legends of figures 4C, G; EV6 B

Please let me know if you have any questions regarding any of these points. You can use the link below to upload the revised files.
Thank you again for giving us the chance to consider your manuscript for The EMBO Journal. I look forward to receiving the final

version.
With best regards,
Cornelius Schneider

____

Cornelius Schneider, PhD
Editor | The EMBO Journal
c.schneider@embojournal.org

Please refer to our figure preparation guideline in order to ensure proper formatting and readability in print as well as on screen:

https://bit.ly/EMBOPressFigurePreparationGuideline

See also figure legend guidelines:

https://www.embopress.org/page/journal/14602075/authorguide#figureformat

Use the link below to submit your revision:

https://emboj.msubmit.net/cgi-bin/main.plex

Referee #1:

The authors addressed our comments to our satisfaction. This is a very nice paper and we support its publication. Two minor revisions should still be done before publication:
1) Fig. EV3B, C is wrongly named Fig. S3B, C
2) Figure 6A was not redesigned with the requested information added as stated in the response; this should be corrected.

Referee #2:

I have seen the figure and found that the data provided in the new panels are sufficient to address my concerns.

All editorial and formatting issues were resolved by the authors.

2nd Revision - Editorial Decision 29th Oct 2025

Dear Dr. Taliaferro,

I am pleased to inform you that your manuscript has been accepted for publication in the EMBO Journal.

Your manuscript will be processed for publication by EMBO Press. It will be copy edited and you will receive page proofs prior to publication. Please note that you will be contacted by Springer Nature Author Services to complete licensing and payment information.

You may qualify for financial assistance for your publication charges - either via a Springer Nature fully open access agreement or an EMBO initiative. Check your eligibility: https://www.embopress.org/page/journal/14602075/authorguide#chargesguide

Should you be planning a Press Release on your article, please get in contact with embo_production@springernature.com as early as possible in order to coordinate publication and release dates.

If you have any questions, please do not hesitate to contact the Editorial Office. Thank you for your contribution to The EMBO Journal.

Yours sincerely,

Cornelius Schneider, PhD
Editor
The EMBO Journal
c.schneider@embojournal.org

*****
Please note that it is The EMBO Journal policy for the transcript of the editorial process (containing referee reports and your response letters) to be published as an online supplement to each paper. If you should prefer removal of any referee-only figures included in the point-by-point response(s), e.g. because they may still be used for future publication or because they have been reproduced from published work by others, please do let us know immediately via response email.
More information is available here: https://www.embopress.org/transparent-process#Review_Process
*****

** Click here to be directed to your login page: https://emboj.msubmit.net

EMBO Press Author Checklist

Corresponding Author Name: Matthew Taliaferro
Journal Submitted to: The EMBO Journal
Manuscript Number: EMBOJ-2025-121163

USEFUL LINKS FOR COMPLETING THIS FORM
The EMBO Journal - Author Guidelines
EMBO Reports - Author Guidelines
Molecular Systems Biology - Author Guidelines
EMBO Molecular Medicine - Author Guidelines

Reporting Checklist for Life Science Articles (updated January
This checklist is adapted from Materials Design Analysis Reporting (MDAR) Checklist for Authors. MDAR establishes a minimum set of requirements in transparent reporting in the life sciences (see Statement of Task: 10.31222/osf.io/9sm4x). Please follow the journal's guidelines in preparing your manuscript.
Please note that a copy of this checklist will be published alongside your article.

Abridged guidelines for figures
1. Data
The data shown in figures should satisfy the following conditions:
- the data were obtained and processed according to the field's best practice and are presented to reflect the results of the experiments in an accurate and unbiased manner.
- ideally, figure panels should include only measurements that are directly comparable to each other and obtained with the same assay.
- plots include clearly labeled error bars for independent experiments and sample sizes. Unless justified, error bars should not be shown for technical
- if n<5, the individual data points from each experiment should be plotted. Any statistical test employed should be justified.
- Source Data should be included to report the data underlying figures according to the guidelines set out in the authorship guidelines on Data

2. Captions
Each figure caption should contain the following information, for each panel where they are relevant:
- a specification of the experimental system investigated (eg cell line, species name).
- the assay(s) and method(s) used to carry out the reported observations and measurements.
- an explicit mention of the biological and chemical entity(ies) that are being measured.
- an explicit mention of the biological and chemical entity(ies) that are altered/varied/perturbed in a controlled manner.
- the exact sample size (n) for each experimental group/condition, given as a number, not a range;
- a description of the sample collection allowing the reader to understand whether the samples represent technical or biological replicates (including how many animals, litters, cultures, etc.).
- a statement of how many times the experiment shown was independently replicated in the laboratory.
- definitions of statistical methods and measures:

- common tests, such as t-test (please specify whether paired vs. unpaired), simple χ2 tests, Wilcoxon and Mann-Whitney tests, can be unambiguously identified by name only, but more complex techniques should be described in the methods section;

- are tests one-sided or two-sided?
- are there adjustments for multiple comparisons?
- exact statistical test results, e.g., P values = x but not P values < x;
- definition of 'center values' as median or average;
- definition of error bars as s.d. or s.e.m.

Please complete ALL of the questions below.
Select "Not Applicable" only when the requested information is not relevant for your study.

Materials

Newly Created Materials	Information included in the manuscript?	In which section is the information available? (Reagents and Tools Table, Materials and Methods, Figures, Data Availability Section)
New materials and reagents need to be available; do any restrictions apply?	Yes	Reagents and tools table

Antibodies	Information included in the manuscript?	In which section is the information available? (Reagents and Tools Table, Materials and Methods, Figures, Data Availability Section)
For **antibodies** provide the following information:<br>- Commercial antibodies: RRID (if possible) or supplier name, catalogue number and or/clone number<br>- Non-commercial: RRID or citation	Yes	Reagents and tools table

DNA and RNA sequences	Information included in the manuscript?	In which section is the information available? (Reagents and Tools Table, Materials and Methods, Figures, Data Availability Section)
**Short novel DNA or RNA including primers, probes**: provide the sequences.	Yes	Reagents and tools table

Cell materials	Information included in the manuscript?	In which section is the information available? (Reagents and Tools Table, Materials and Methods, Figures, Data Availability Section)
**Cell lines:** Provide species information, strain. Provide accession number in repository **OR** supplier name, catalog number, clone number, and/**OR** RRID.	Yes	Reagents and tools table and Materials and Methods
**Primary cultures:** Provide species, strain, sex of origin, genetic modification status.	Not Applicable	
Report if the cell lines were recently **authenticated** (e.g., by STR profiling) and tested for mycoplasma contamination.	Yes	Materials and methods

Experimental animals	Information included in the manuscript?	In which section is the information available? (Reagents and Tools Table, Materials and Methods, Figures, Data Availability Section)
**Laboratory animals or Model organisms:** Provide species, strain, sex, age, genetic modification status. Provide accession number in repository **OR** supplier name, catalog number, clone number, **OR** RRID.	Not Applicable	
**Animal observed in or captured from the field:** Provide species, sex, and age where possible.	Not Applicable	
Please detail **housing and husbandry conditions**.	Not Applicable	

Plants and microbes	Information included in the manuscript?	In which section is the information available? (Reagents and Tools Table, Materials and Methods, Figures, Data Availability Section)
**Plants:** provide species and strain, ecotype and cultivar where relevant, unique accession number if available, and source (including location for collected wild specimens).	Not Applicable	
**Microbes:** provide species and strain, unique accession number if available, and source.	Not Applicable	

Human research participants	Information included in the manuscript?	In which section is the information available? (Reagents and Tools Table, Materials and Methods, Figures, Data Availability Section)
If collected and within the bounds of privacy constraints report on age, sex and gender or ethnicity for all study participants.	Not Applicable	

Core facilities	Information included in the manuscript?	In which section is the information available? (Reagents and Tools Table, Materials and Methods, Figures, Data Availability Section)
If your work benefited from core facilities, was their service mentioned in the acknowledgments section?	Not Applicable	

Design

Study protocol	Information included in the manuscript?	In which section is the information available? (Reagents and Tools Table, Materials and Methods, Figures, Data Availability Section)

If study protocol has been **pre-registered, provide DOI in the manuscript**. For clinical trials, provide the trial registration number **OR** cite DOI.	Not Applicable	
Report the **clinical trial registration number** (at ClinicalTrials.gov or equivalent), where applicable.	Not Applicable	

Laboratory protocol	Information included in the manuscript?	In which section is the information available? (Reagents and Tools Table, Materials and Methods, Figures, Data Availability Section)
Provide DOI OR other citation details if **external detailed step-by-step protocols** are available.	Yes	Subcellular fractionation reference (Arora A, Goering R, Lo H-YG & Taliaferro MJ (2021a) Mechanical fractionation of cultured neuronal cells into cell body and neurite fractions. Bio Protoc 11: e4048) in Materials and Methods

Experimental study design and statistics	Information included in the manuscript?	In which section is the information available? (Reagents and Tools Table, Materials and Methods, Figures, Data Availability Section)
Include a statement about **sample size** estimate even if no statistical methods were used.	Yes	Subcellular fractionation experiments were performed in triplicate (Figure EV1F,G). MPRA experiments were performed in quadruplicate (Figure EV3B). RBNS experiments were performed in duplicate (Figure EV4A). SLAM-seq was performed in triplicate (Materials and methods)
Were any steps taken to minimize the effects of subjective bias when allocating animals/samples to treatment (e.g. **randomization procedure**)? If yes, have they been described?	Not Applicable	
Include a statement about **blinding** even if no blinding was done.	Not Applicable	
Describe **inclusion/exclusion criteria** if samples or animals were excluded from the analysis. Were the criteria pre-established?<br><br>If sample or data points were omitted from analysis, report if this was due to attrition or intentional exclusion and provide justification.	Not Applicable	
For every figure, are **statistical tests** justified as appropriate? Do the data meet the assumptions of the tests (e.g., normal distribution)? Describe any methods used to assess it. Is there an estimate of variation within each group of data? Is the variance similar between the groups that are being statistically compared?	Yes	For almost all tests, nonparametric tests (e.g. Wilcoxon) were used. For tests with small numbers of observations (e.g. qPCR experiments in figures 3D and 4C), T-tests were used. The identity of all tests used are indicated in the figure legends.

Sample definition and in-laboratory replication	Information included in the manuscript?	In which section is the information available? (Reagents and Tools Table, Materials and Methods, Figures, Data Availability Section)
In the figure legends: state number of times the experiment was **replicated** in laboratory.	Yes	As stated above: Subcellular fractionation experiments were performed in triplicate (Figure EV1F,G). MPRA experiments were performed in quadruplicate (Figure EV3B). RBNS experiments were performed in duplicate (Figure EV4A). SLAM-seq was performed in triplicate (Materials and methods).
In the figure legends: define whether data describe **technical or biological replicates**.	Yes	Figure legends

Ethics

Ethics	Information included in the manuscript?	In which section is the information available? (Reagents and Tools Table, Materials and Methods, Figures, Data Availability Section)
Studies involving **human participants**: State details of **authority granting ethics approval** (IRB or equivalent committee(s), provide reference number for approval.	Not Applicable	
Studies involving **human participants**: Include a statement confirming that **informed consent** was obtained from all subjects and that the experiments conformed to the principles set out in the WMA Declaration of Helsinki and the Department of Health and Human Services Belmont Report.	Not Applicable	
Studies involving **human participants:** For publication of **patient photos**, include a statement confirming that consent to publish was obtained.	Not Applicable	
Studies involving experimental **animals**: State details of **authority granting ethics approva**l (IRB or equivalent committee(s), provide reference number for approval. Include a statement of compliance with ethical regulations.	Not Applicable	
Studies involving **specimen and field samples:** State if relevant **permits** obtained, provide details of authority approving study; if none were required, explain why.	Not Applicable	

Dual Use Research of Concern (DURC)	Information included in the manuscript?	In which section is the information available? (Reagents and Tools Table, Materials and Methods, Figures, Data Availability Section)
Could your study fall under dual use research restrictions? Please check biosecurity documents and list of **select agents and toxins** (CDC): https://www.selectagents.gov/sat/list.htm	Not Applicable	
If you used a select agent, is the security level of the lab appropriate and reported in the manuscript?	Not Applicable	
If a study is subject to dual use research of concern regulations, is the name of the **authority granting approval and reference number** for the regulatory approval provided in the manuscript?	Not Applicable	

Reporting

The MDAR framework recommends adoption of discipline-specific guidelines, established and endorsed through community initiatives. Journals have their own policy about requiring specific guidelines and recommendations to complement MDAR.

Adherence to community standards	Information included in the manuscript?	In which section is the information available? (Reagents and Tools Table, Materials and Methods, Figures, Data Availability Section)
State if relevant guidelines or checklists (e.g., **ICMJE, MIBBI, ARRIVE, PRISMA**) have been followed or provided.	Not Applicable	
For **tumor marker prognostic studies**, we recommend that you follow the **REMARK** reporting guidelines (see link list at top right). See author guidelines, under 'Reporting Guidelines'. Please confirm you have followed these guidelines.	Not Applicable	
For **phase II and III randomized controlled trials**, please refer to the **CONSORT** flow diagram (see link list at top right) and submit the CONSORT checklist (see link list at top right) with your submission. See author guidelines, under 'Reporting Guidelines'. Please confirm you have submitted this list.	Not Applicable	

Data Availability

Data availability	Information included in the manuscript?	In which section is the information available? (Reagents and Tools Table, Materials and Methods, Figures, Data Availability Section)
Have **primary datasets** been deposited according to the journal's guidelines (see 'Data Deposition' section) and the respective accession numbers provided in the Data Availability Section?	Yes	All seqeuencing data has been deposited in the Gene Expression Omnibus under accession number GSE288185. This is indicated in the Data Availability Section.
Were **human clinical and genomic datasets** deposited in a public access-controlled repository in accordance to ethical obligations to the patients and to the applicable consent agreement?	Not Applicable	
Are **computational models** that are central and integral to a study available without restrictions in a machine-readable form? Were the relevant accession numbers or links provided?	Not Applicable	
If publicly available data were reused, provide the respective **data citations in the reference list**.	Yes	Reused publically available data has been cited in the reference list.
